# New Tools to Study Contact Activation

**DOI:** 10.3389/fmed.2016.00058

**Published:** 2016-11-22

**Authors:** Steffen Rosén

**Affiliations:** ^1^Private Practice, Molndal, Sweden

**Keywords:** FXIa, FXIIa, kallikrein, methods, chromogenic

## Abstract

The recent availability of a sensitive chromogenic method approach for determination of FXIa activity has been explored for designing sensitive methods for FXIIa and kallikrein, both using FXa formation as the read-out. For both enzymes the assay range 1–10 nmol/L provides a resolution of about 0.8 absorbance units with a total assay time of about 20 min. For studies on activation kinetics, subsampling and extensive dilution can be performed in MES–bovine serum albumin (BSA) buffer pH 5.7 for quenching of enzyme activity and with ensuing determination of FXa generation in a chromogenic FXIa method. Optionally, suitable inhibitors such as aprotinin and/or corn trypsin inhibitor may be included. The stability of FXIa, FXIIa, and kallikrein in MES–BSA buffer was shown to be at least 5 h on ice. In conclusion, the use of a sensitive chromogenic FXIa method either per se or in combination with MES–BSA buffer pH 5.7 are new and potentially valuable tools for the study of contact factor enzymes and their inhibitors. So far, dose–response studies of FXIIa and kallikrein have been limited to purified systems, and hence more data are required to learn whether these new methods might or might not be applicable to the determination of FXIIa and kallikrein activities in plasma.

## Introduction

The contact activation pathway, involving factor XII (FXII), prekallikrein (PK), high molecular weight kininogen (HK), and factor XI (FXI) displays complex interactions and is involved in both coagulation and inflammation [for reviews, see Ref. (1, 2)]. Much research has been devoted to this area during the last 20 years, partly due to the findings that FXI can be activated on the platelet surface by thrombin (3–8) and also due to the more recent interest in anti-FXI antibodies and FXI antisense nucleotides as antithrombotic agents (9–13).

*In vivo* as well as for *in vitro* studies on plasma, contact activation is surface-bound, i.e., contact factors assemble on a negatively charged surface with the initial step being binding and autoactivation of FXII. PK and FXI bind to the surface through HK. *In vitro*, this is utilized in any APTT-based method, and the negatively charged surface is provided as a constituent in the APTT reagent, typically as some variant of silica or as ellagic acid.

Studies performed more than 40 years ago demonstrated that surface-bound FXII and FXI are much more effectively activated than in solution and also that HK is a cofactor to such activation, however not a mandatory requirement (14–16).

More recently, sensitive chromogenic methods for FXIa have been developed and made available as commercial kits (17, 18). These products were originally developed for detection of trace amounts of FXIa as contaminant in pharmaceutical products such as intravenous immunoglobulins. However, the chromogenic FXIa method has also been applied for investigation of FXI activation during contact activation on analysis of a one-stage method for potency assignment of the extended half-life rFIX product N9-GP (19). Here, subsampling and dilution into a low pH buffer was used to quench further activation. This combined approach has now been explored for designing sensitive methods for FXIIa and kallikrein. Part of this work was presented at the ISTH/SSC meeting in May 2016 (20).

## Materials and Methods

Chromogenic kit Rox Factor XIa (Rossix AB, Molndal, Sweden) was used for photometric determination of FXIa activity. The kit comprises two lyophilized reagents, one containing human factor IX, human factor VIII, and calcium chloride and the other containing human factor X, bovine thrombin, phospholipids, and calcium chloride. The chromogenic FXa substrate Carboxybenzyl-D-Arg-Gly-Arg-pNA is included in the kit as a liquid solution. The limit of quantitation in the assay is about 0.02 mIU/mL, calibrated vs. the WHO first Int Std FXIa (ref 13/100). This corresponds roughly to 0.01 pmol/L of FXIa.

Bovine serum albumin (BSA) was approved for use as a bulking agent of coagulation proteins (Rossix AB).

The coagulation proteins human FXI, human FXIa, human FXII, human αFXIIa, human PK, human plasma kallikrein (kallikrein), and corn trypsin inhibitor (CTI) were all from Enzyme Research Laboratories (South Bend, IN, USA).

Frozen human normal pooled plasma (NPL) was from Precision Biologics (Dartmouth, Canada).

Activated partial thromboplastin reagent APTT SP was from Instrumentation Laboratory, Bedford, MA, USA.

Bovine lung aprotinin was from Sigma-Aldrich, St Louis, MI, USA.

Tris(hydroxymethyl)aminomethane (Tris), Tris hydrochloride, 2-(N-morpholino)ethanesulfonic acid (MES), and sodium chloride were from Sigma-Aldrich. All chemicals were of reagent grade.

MES–BSA buffer was prepared to contain 0.05 mol/L MES pH 5.7, 0.05 M NaCl, 0.2% BSA.

Also, 0.1 mol/L Tris buffer pH 8.3 (25°C) was used for neutralization in assays with samples in MES buffer.

Dilution of coagulation proteins was made in 0.05 mol/L Tris buffer pH 7.5 (25°C), 0.1 mol/L NaCl, 1% BSA, denoted working buffer solution (WBS).

### Determination of FXIa Activity

FXIa activity was determined with the Rox Factor XIa kit method. The activity is expressed in milli-international units per milliliter.

### Determination of FXIIa Activity through Generation of FXIa

At different doses, 25 μL FXIIa was mixed in microplate wells with 50 μL FXI and 25 μL WBS, whereafter 25 μL APTT SP was added to provide a contact activation surface. Final concentrations were 0, 1–10 nmol/L of FXIIa, and 40 nmol/L of FXI.

The activation of FXI was allowed to proceed for 6 min at 37°C followed by addition of 50 μL CTI (final concentration 0.35 μM) for inactivation of FXIIa. After a further incubation for 2 min at 20–25°C to ensure complete inactivation of FXIIa, simultaneous consecutive dilutions of all incubation mixtures were made in MES–BSA buffer in microplate wells to reach a 250-fold dilution.

Then, generated FXIa activity was determined with the Rox Factor XIa kit method and with supplementation of 0.1 mol/L Tris buffer pH 8.3 for neutralization.

### Determination of Kallikrein Activity through Generation of FXIa *via* FXII Activation

At different doses, 25 μL kallikrein was mixed in microplate wells with 50 μL FXI, 8 μL FXII, and 25 μL WBS whereafter 25 μL APTT SP was added to provide a contact activation surface. Final concentrations were 0, 1–10 nmol/L of kallikrein, 40 nmol/L of FXI, and 93 nmol/L of FXII.

The activation of FXII and FXI was allowed to proceed for 6 min at 37°C followed by addition of 50 μL CTI (final concentration 4 μg/mL) for inactivation of FXIIa. After a further incubation for 2 min at 20–25°C to ensure complete inactivation of FXIIa, simultaneous consecutive dilutions of all incubation mixtures were made in MES–BSA buffer in microplate wells to reach 50- and 200-fold dilutions.

Then, generated FXIa activity was determined with the Rox Factor XIa kit method and with supplementation of 0.1 mol/L Tris buffer pH 8.3 for neutralization.

### Stability Tests in MES–BSA Buffer

The above biological activity methods for FXIa, FXIIa, and kallikrein were utilized to study the stability of these enzymes in MES–BSA buffer. WBS was first diluted fourfold in water to decrease its buffering capacity and then used for suitable predilution of each enzyme. These were subsequently 21-fold diluted in ice cold MES–BSA buffer and kept on ice. Also, 50 μL aliquots were withdrawn from each enzyme solutions at time points 0, 1, 2, 3, 4, and 5 h and immediately frozen at −70°C and kept at −70°C until analysis. The biological activities of FXIa, FXIIa, and kallikrein were then determined as described above.

### Determination of Stability of FXIa Added to Plasma or Generated in Plasma *via* Contact Activation

#### Contact-Activated FXI

Three hundred and thirty microliter NPL was contact activated with 165 μL APTT SP for 5 min, followed by addition of 20 μL of CTI (final concentration 4 μg/mL) and 20 μL of aprotinin (final concentration 350 KIU/mL) to inhibit FXIIa and kallikrein, respectively. The concentration used of aprotinin inhibited >98% of kallikrein activity and about 70% of FXIa activity.

A first subsampling was then immediately made of 10 μL into 3 mL MES–BSA buffer (301-fold dilution) followed by further subsamplings after 30 s and up to 20 min.

#### FXIa Spiked into Plasma

Twenty microliter FXIa was added to 480 μL human normal plasma and subsampling was made of 10 μL into 3 mL MES–BSA buffer after 30 s followed by further subsamplings up to 20 min. The zero time sample was obtained by adding 10 μL of a 25-fold lower FXIa concentration directly to 3 mL MES–BSA buffer.

The FXIa activity was then determined as described above.

## Results

### Dose–Response Curves for FXIa, FXIIa, and Kallikrein

**Figure 1:**
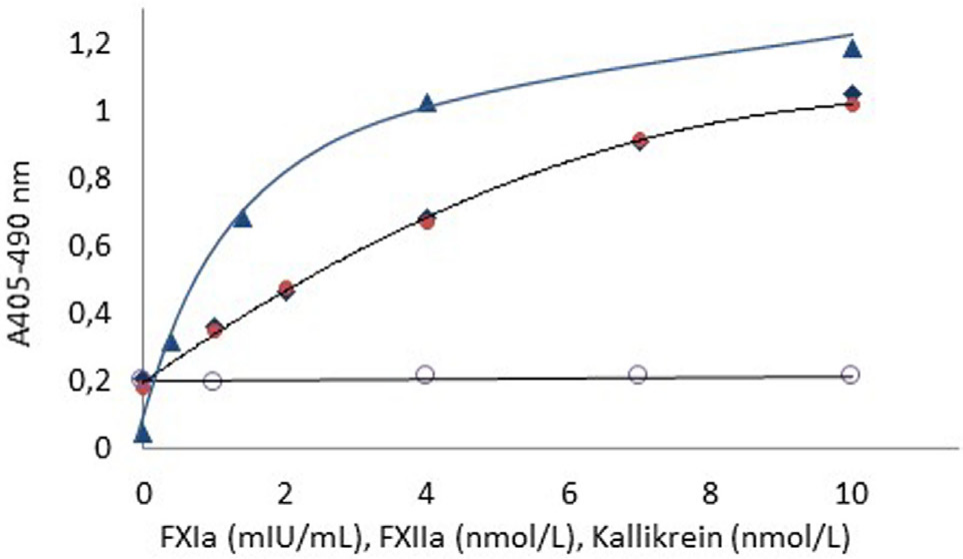
**Dose–response curves for FXIa (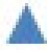), FXIIa (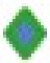), and kallikrein (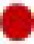) using APTT SP as contact surface provider**. The dose–response curve for kallikrein in the absebce of APTT SP (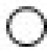) is also shown. The higher blank value for FXIIa and kallikrein as compared to FXIa is due to presence of FXIa in the FXI zymogen preparation.

Figure 1 shows dose–response curves for FXIa (1–10 mIU/mL), FXIIa (1–10 nmol/L), and kallikrein (1–10 nmol/L) using FXa formation as the read-out with the Rox Factor XIa kit and hence expressing biological functional activities of these enzymes.

For all enzymes, the resolution was at least 0.8 absorbance units in the tested ranges. The blank activity of about 0.2 absorbance units noticed for the FXIIa and kallikrein curves is due to contaminating FXIa in the used FXI zymogen preparation.

### Stability of FXIa, FXIIa, and Kallikrein in MES–BSA Buffer

The stability of FXIa, FXIIa, and kallikrein was determined after storage for up to 5 h in ice cold MES–BSA buffer pH 5.7 using the above methods. For all enzymes, the recovery of activity was between 95 and 102% of the initial activity during the 5-h storage time. Similar results were also obtained from determination of enzyme activities with direct hydrolysis of suitable chromogenic substrates (data not shown).

### Stability of FXIa Added to Plasma or Generated in Plasma *via* Contact Activation

**Figure 2:**
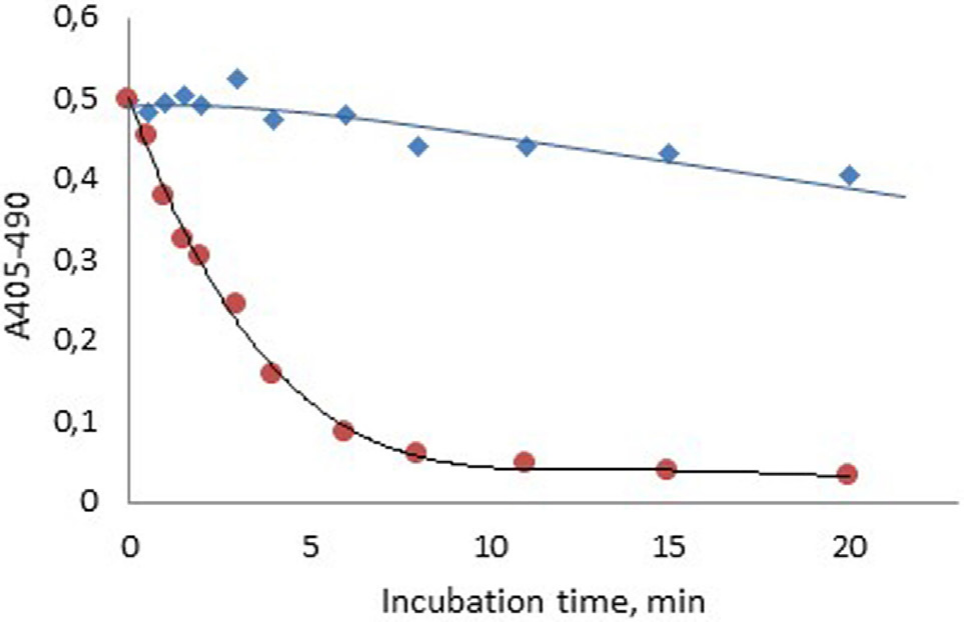
**Stability of human FXIa in normal plasma after formation *via* contact activation with APTT SP (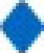) or after spiking into plasma (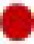)**.

Figure 2 shows that about 85% of the FXIa activity was lost 6 min after spiking of FXIa to plasma, whereas about 96% of the FXIa activity remained after 6 min when generated during contact activation. The activity then fell gradually but was still above 80% after 20 min storage.

## Discussion

The high sensitivity of the chromogenic FXIa method (0.02 mIU/mL or about 0.01 pmol/L) allows extensive sample dilutions to minimize or eliminate interferences from sample matrices. This has been utilized in the present study for designing biological activity methods also for FXIIa and kallikrein as shown in Figure 1. Such methods reflect the functional, biological activity in contrast to direct amidolytic methods in which suitable chromogenic substrates are directly cleaved by FXIa, FXIIa, and kallikrein. Such methods may not always reflect the biological activity as is illustrated, e.g., by γ-thrombin, which cleaves low molecular chromogenic substrates but splits its natural substrate fibrinogen very poorly (21). Furthermore, enzymes in complex with α_2_-macroglobulin usually cleave chromogenic substrates but not natural protein substrates (22–25).

The FXIIa and kallikrein methods should be regarded as “principle approaches,” demonstrating their feasibility but not claimed to be optimized. Thus, doubling the FXII zymogen concentration in the kallikrein method gave a significant increased rate of FXII activation by kallikrein, and hence an increase in FXIa generation resulting in about 50% higher FXa formation (data not shown). The utilized concentration range, 1–10 nmol/L for FXIIa and kallikrein, corresponds to about 0.25–2.5% and 0.18–1.8% of the plasma zymogen concentrations of FXII and PK, respectively. The methods may tentatively be further amplified by including also the prothrombinase complex in the final step and thus use thrombin formation rather than generation of FXa as the read-out.

Common for all three methods is the use of a contact activator (APTT SP) as a surface provider and trigger of activation. Importantly, HK does not have to be present in agreement with earlier findings (16).

It is also worth mentioning that the used concentrations of CTI and aprotinin only cause ≤5% inhibition of the FXa generation (data not shown).

The demonstrated stability of FXIa, FXIIa, and kallikrein in MES–BSA buffer pH 5.7 may be conveniently utilized in, e.g., studies on activation kinetics where subsampling and extensive dilutions in MES–BSA buffer followed by chromogenic FXIa determination provides a sensitive and robust tool for such studies (19).

This combined concept was also utilized in the present study to demonstrate a pronounced difference in FXIa stability when formed during contact activation in plasma as compared to addition of FXIa in plasma (Figure 2). The higher stability of FXIa in the former case is due to protection of FXIa by HK in the contact factor complex (26). The slow inhibition of generated FXIa, with about 20% inhibition after 20 min is caused by several protease inhibitors, primarily C1-esterase inhibitor, α_2_-antiplasmin, and α_1_-antitrypsin (27).

A further illustration on the advantage with the high sensitivity of the FXIa method is the use of aprotinin for efficient inhibition of kallikrein. Even though the used concentration, 350 KIU/mL, of aprotinin caused about 70% inhibition of FXIa, sufficient FXa generation in the chromogenic FXIa method was easily obtained.

In conclusion, the use of a sensitive chromogenic FXIa method either *per se* or in combination with MES–BSA buffer pH 5.7 may be valuable tools for study of contact factor enzymes and their inhibitors.

The system may tentatively be further amplified by using thrombin formation rather than generation of FXa as the end-point.

## Summary

The recent availability of a sensitive chromogenic method approach for the determination of FXIa activity has been explored for designing sensitive methods for FXIIa and kallikrein, both using FXa formation as the read-out. For both enzymes the assay range 1–10 nmol/L provides a resolution of about 0.8 absorbance units with a total assay time of about 20 min.

For studies on activation kinetics, subsampling and extensive dilution can be performed in MES–BSA buffer pH 5.7 for quenching of enzyme activity, optionally including aprotinin and/or CTI, and with ensuing determination of FXa generation in the chromogenic FXIa method.

The stability of FXIa, FXIIa, and kallikrein in MES–BSA buffer was shown to be at least 5 h on ice.

In conclusion, the use of a sensitive chromogenic FXIa method either *per se* or in combination with MES–BSA buffer pH 5.7 are new and potentially valuable tools for study of contact factor enzymes and their inhibitors. It should be noted that these assays have not been evaluated for the determination of FXIIa and kallikrein in patients’ plasma.

## Author Contributions

SR designed and performed the experiments and wrote the manuscript.

## Conflict of Interest Statement

SR is a consultant to Rossix AB.
